# Trilateral Multi-Functional Polyamide 12 Nanocomposites with Binary Inclusions for Medical Grade Material Extrusion 3D Printing: The Effect of Titanium Nitride in Mechanical Reinforcement and Copper/Cuprous Oxide as Antibacterial Agents

**DOI:** 10.3390/jfb13030115

**Published:** 2022-08-04

**Authors:** Nectarios Vidakis, Markos Petousis, Nikolaos Mountakis, Apostolos Korlos, Vassilis Papadakis, Amalia Moutsopoulou

**Affiliations:** 1 Mechanical Engineering Department, Hellenic Mediterranean University, Estavromenos, 71410 Heraklion, Greece; 2 Department of Industrial Engineering and Management, International Hellenic University, 14th km, Thessaloniki-N. Moudania, Thermi, 57001 Thessaloniki, Greece; 3 Institute of Molecular Biology and Biotechnology, Foundation for Research and Technology—Hellas, 71110 Heraklion, Greece

**Keywords:** polyamide 12 (PA12), titanium nitride (TiN), copper (Cu), cuprous oxide (Cu_2_O), nanocomposites, material extrusion (MEX), 3D printing, mechanical characterization

## Abstract

In this work, for the first time, polyamide 12 (PA12) nanocomposites with binary inclusions in material extrusion (MEX) 3D printing were developed. The aim was to achieve an enhanced mechanical response with the addition of titanium nitride (TiN) and antibacterial performance with the addition of copper (Cu) or cuprous oxide (Cu_2_O), towards the development of multi-functional nanocomposite materials, exploiting the 3D printing process benefits. The prepared nanocomposites were fully characterized for their mechanical properties. The thermal properties were also investigated. Morphological characterization was performed with atomic force microscopy (AFM) and scanning electron microscopy (SEM). The antibacterial performance was investigated with an agar-well diffusion screening process. Overall, the introduction of these nanofillers induced antibacterial performance in the PA12 matrix materials, while at the same time, the mechanical performance was significantly increased. The results of the study show high potential for expanding the areas in which 3D printing can be used.

## 1. Introduction

Polyamides belong to the polymers’ family of materials. They are used in various engineering applications, such as catalytic applications [1], films and membranes [2], food packaging [3] among other fields, and medical applications [4,5], as they come in medical grades [5] and composite forms which are suitable for antibacterial activities [6] and orthopedic applications [7]. Polyamide 12 (PA12) is a popular polyamide, due to its enhanced thermal and mechanical response [8]. This specific grade has been investigated and applied in different additive manufacturing (AM) processes, such as binder jetting [9], powder bed fusion [10,11], and material extrusion (MEX) as pure material, or as the matrix material in composites [5,8,12,13,14,15,16,17,18,19,20,21]. PA12 is a material suitable for the 3D printing (3DP) process, due to its rheological characteristics [22], its toughness, and high strain values before its failure [10,23,24,25]. 3D printing orientation is also a critical parameter affecting the mechanical properties of the built parts [26], and this applies to the PA12 polymer as well [27].

Additionally, it is an eco-friendly material, that can be processed at least five times with a thermomechanical procedure and maintain its stability and its mechanical response [22]. Finally, PA12 can be processed with additives for the development of composites and nanocomposites for the enhancement of its mechanical response, or to induce a multi-functional characteristic to the material [5,25,28].

AM fields of application are increasing nowadays, leading to an increased demand for materials in AM with corresponding enhanced properties. The main drawback of the 3DP parts is the reduced mechanical properties compared to the injection-molded parts [29,30,31]. To overcome this issue, various types of nanomaterials have been introduced into polymer matrices that enhance the mechanical response of the matrix material and induce their properties in the developed nanocomposites [5,20,31,32]. As a result, nanocomposites for 3DP and AM, in general, have been developed and characterized with improved mechanical, electrical, and thermal properties [20,33,34]. At the same time, properties such as antibacterial response have been introduced in biocompatible and medical grade materials, making them suitable for different healthcare and medical applications [4,5,8,35].

Carbides and nitrides are known for their advanced mechanical properties, and therefore they are used in various forms as parts. They operate in demanding environments, such as in cutting tools [36], as coatings [37], due to their hardness [38], in titanium implants to enhance the mechanical properties of the implant [39], and in nanoscale and nanofiber form as an additive to enhance the mechanical properties of the materials [40,41]. Titanium nitride (TiN) is a nitride used in different types of applications, such as optical applications [42,43,44,45,46], coatings and films due to its wear resistance capabilities [47,48,49], energy and electrical applications [50,51], and in healthcare applications [52]. In some of these technological fields, TiN has been integrated into the AM process, i.e., in optical applications [53], coatings [54], and medical implants [55]. TiN has been used in nanopowder form for the enhancement of the mechanical properties of the Polycarbonate (PC) thermoplastic in MEX 3DP [56]. Research in TiN with polyamides is focusing mainly on its use as a coating for the PA6 [57,58]. TiN as an additive for enhancing the mechanical properties of the PA12 has not been presented in the literature so far, for MEX 3DP or any other manufacturing process.

For the development of composites and nanocomposites with antibacterial properties, additives with such properties are introduced into the materials, with copper (Cu) and its oxides, such as copper (I) oxide (cuprous oxide, Cu_2_O), frequently used in the literature [8,32,34,35]. Cu in different types (nanopowder and others) has been widely used and investigated in the literature for various types of applications [59,60,61,62]. It has been processed and investigated in AM as well [60,61,63,64,65,66], with several works exploiting its antibacterial performance in AM [34,65]. Cuprous oxide has been employed in semiconductors [67,68], among other applications; still, it is mainly used and investigated for its antibacterial properties [69,70,71,72,73,74,75,76]. Its antibacterial properties have also been exploited in vat photopolymerization [32,34] and MEX 3DP for polylactic acid (PLA) [35] and PA12 polymers [8], achieving sufficient antibacterial performance in the nanocomposites developed in these studies. Copper and other metal nanoparticles (NPs) biocidal properties are achieved, since they have physicochemical characteristics, imitating the host defense peptide (HDP), which is the main mechanism in living organisms for killing or inhibiting bacteria [77,78]. The exact mechanism of these NPs in killing bacteria is still not well known [79].

A multi-functional performance of the material is an asset in various applications, especially in the medical field, for example in medical devices, in which enhanced mechanical performance is required along with antibacterial behavior [80]. In this work, to achieve such performance, nanocomposites were developed with binary inclusions for MEX 3DP. No similar study is available in the literature so far which combines these specific materials and exploits 3DP technology. These materials were selected to be investigated, due to their characteristics and their wide field of application, as analyzed in the literature review above about them. An additive was used (TiN) to enhance the mechanical performance and different additives were also used (Cu or Cu_2_O) to induce antibacterial properties in the developed nanocomposites. Polyamide 12 (PA12) was the matrix material in the work. Materials were prepared with a thermomechanical process, in a form suitable for MEX 3DP. The 3DP samples were evaluated for their thermal, mechanical, and antibacterial performance. Their morphological characteristics and the fracture mechanism of the tensile samples were investigated with atomic force microscopy (AFM) and scanning electron microscopy (SEM). Overall, the aim of this work was achieved, with the prepared nanocomposites exhibiting a radically improved mechanical response compared to the pure PA12 polymer, while all the Cu and Cu_2_O nanocomposites had a biocidal performance for the two bacteria assessed (gram-negative Escherichia coli—*E. coli*, and gram-positive Staphylococcus aureus—*S. Aureus*) with the agar-well diffusion screening process. Such results show the potential of the process followed for the development of multi-functional nanocomposites, exploiting the benefits of the MEX 3DP process and further expanding its fields of application.

## 2. Materials and Methods

**Figure 1:**
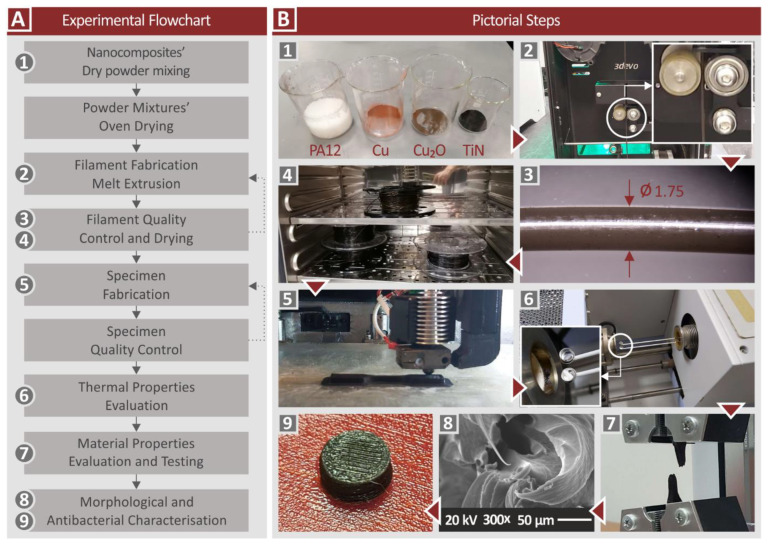
Workflow of the current study.

Figure 1 presents this study’s workflow.

### 2.1. Materials Used for the Preparation of the Study’s Nanocomposites

The matrix material in the nanocomposites prepared in this work was polyamide 12 (PA12). It was sourced in fine granules from Arkema (Colombes, France). The type was Rilsamid PA12 AESNO TL. This was a medical grade PA12, with improved heat and UV stabilization, due to its low percentage of additives, according to the technical datasheet. It further had the following specifications: melting temperature of 180 °C (ISO 11357-3), melt volume–flow rate (MVR) of 8.0 cm^3^/10 min (ISO 1133) at 235 °C/5.0 kg, density 1.01 g/cm^3^ (ISO 1183), and Vicat softening temperature at 142 °C (ISO 306/B50). Although this was medical-grade PA12, it should not be used in implants, or be in contact with body tissues for more than 30 days.

For the enhancement of the mechanical performance of the PA12 polymer, Titanium nitride (TiN) was used. It was sourced in nanopowder form, from Nanographi (Ankara, Turkey). Its technical specifications were size 20 nm, shape cubic, purity 99.2+%, true density 5.3 gr/cm^3^, melting point 2950 °C, and specific Surface Area 50–80 m^2^/gr.

For inducing antibacterial properties, two copper-based additives were assessed, individually one from the other, so different nanocomposites were developed with each additive, to evaluate the antibacterial performance of each additive. Both were sourced from Nanografi (Nanografi Inc., Ankara, Turkey) in nanopowder form. These were Copper nanoparticles (Cu) (purity 99.95%, size 80 nm to 240 nm) and Cuprous Oxide I (Cu_2_O) (purity is 99.5%, size 80 nm).

### 2.2. Preparation of the Nanocomposites

Nanocomposites in this work were prepared with a thermomechanical extrusion process in 1.75 mm filament form, suitable for MEX 3DP. The aim was to develop nanocomposites with binary inclusions, exhibiting enhanced mechanical responses and having antibacterial properties. The effect of each filler in the matrix material was initially investigated, so nanocomposites with PA12 as the matrix material and one of the fillers were prepared. With the TiN additive, nanocomposites with three different weight-to-weight (wt.%) concentrations were prepared, i.e., 1.0, 2.0, and 4.0 wt.%. The effect on the filler loading in the mechanical enhancement of the matrix material was evaluated through this approach. PA12/Cu 0.5 wt.% nanocomposites were prepared to evaluate mainly the effectiveness of the filler in the antibacterial performance of the polymer, but also a possible enhancement in the mechanical properties. Also, corresponding PA12/Cu_2_O 0.5 wt.% nanocomposites were prepared for the same purpose. With this procedure, the effect of each filler on the PA12 matrix was determined. Then, nanocomposites with binary inclusions were prepared, i.e., PA12/TiN/Cu and PA12/TiN/Cu_2_O. The TiN loading selected was the one that exhibited the highest mechanical response (2.0 wt.%), while the antibacterial agents have the same loading as the corresponding single additive nanocomposites. The effect of binary inclusions on the PA12 matrix was evaluated, compared to corresponding single additive nanocomposites. Additionally, the aim of developing multi-functional nanocomposites for MEX 3DP, with improved mechanical properties and antibacterial performance was explored.

Raw materials were first oven-dried (60 °C for 24 h), to remove any possible humidity from them. Then, separate mixtures for each different nanocomposite combination were prepared, in a high-power blender. The procedure took place in a glovebox, to restrain the spreading of the powders in the atmosphere. Mixtures were successively afterward turned into filament on a Noztek (Shoreham-by-Sea, UK) desktop single-screw extruder. The filament was then turned into pellets (3devo shredder, Utrecht, The Netherlands). These pellets were then turned into 1.75 mm filament for MEX 3DP, utilizing a 3devo Composer (Utrecht, The Netherlands) single-screw extruder, specially designed for materials mixing, using the following, experimentally determined, parameters: 4 rpm, fan 95%, nozzle area temperature 231 °C, mid-chamber zones 237 °C and 235 °C, and hopper area temperature 225 °C. This procedure, with the two successive extrusion processes, was followed to achieve the best possible dispersion of the fillers in the nanocomposites. For evaluation purposes, a filament with pure PA12 was also prepared.

### 2.3. Fabrication of the 3D-Printed Specimens

**Figure 2:**
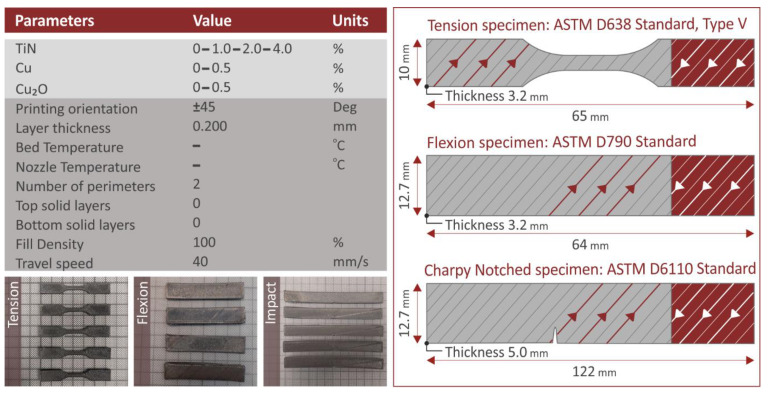
Additives concentration (wt.%) in the nanocomposites, 3D printing settings for their fabrication, and the geometry and infill pattern of the prepared 3D-printed samples in the work. The corresponding standard for each mechanical test was followed.

An Intamsys Funmat HT (Shanghai, China) MEX 3D printer was utilized for the fabrication of the 3D-printed specimens, with the prepared filaments, for the mechanical characterization of the nanocomposites. Five specimens for each mechanical test, following the corresponding standard, were prepared, with the 3D printing settings applied (Figure 2), experimentally determined before the specimens were built. The Intamsuite software platform (Shanghai, China) was used for the required G-codes compilation.

### 2.4. Thermogravimetric Analysis (TGA) and Raman Spectra

Thermogravimetric analysis (TGA) was performed on all the prepared nanocomposites and the pure PA12 polymer, to determine their thermal properties and the effect of the additives on the thermal behavior of the pure PA12. Measurements were taken in a temperature range of 40 °C to 550 °C, with a temperature step of 10 °C/min, in a nitrogen atmosphere. A Perkin Elmer Diamond apparatus (Waltham, MA, USA) apparatus was used and corresponding weight loss and weight loss rate vs. temperature graphs were produced for each material.

Raman measurements were performed with a modified LabRAM HR Raman spectrometer (HORIBA Scientific, Kyoto, Japan). Raman excitation was achieved with a 532 nm central wavelength solid-state laser module with a maximum laser output power of 90 mW. The microscope is coupled with a 50× microscopic objective lens with a 0.5 numerical aperture and 10.6 mm working distance (LMPlanFL N, Olympus, Tokyo, Japan) that delivered the excitation light and collected the Raman signals. A neutral density filter of 5% transmittance was used which resulted in 2 mW of power on the sample. The laser spot size was proximately 1.7 μm laterally, and about 2 μm axially. A 600 groves grating was used resulting in a Raman spectral resolution of around 2 cm^−1^. The Raman spectral range was set to be from 50 to 3900 cm^−1^, resulting in 3 optical windows per point. The acquisition time for each measurement was 10 s and with 3 accumulations at each point.

### 2.5. Filament Quality Control

The quality of the produced filament for all the materials’ combinations in this work was evaluated through a morphological analysis conducted with atomic force microscopy (AFM) (MicroscopeSolver P47H Pro, Moscow, Russia, 300 kHz resonant frequency). The Park SmartScan (Park Systems Corp., Suwon, Korea) software was utilized for the AFM measurements. Measurements were taken on the side surface of the produced filament. Such measurements provide an indication of the filament extrusion process and the filament quality. This filament was then used for the MEX 3D printing of the specimens. The effect of each different additive or additives-combination on the side surface quality of the produced filament was evaluated with this process. The AFM measurements conducted in the work do not follow an International Organization for Standardization (ISO) or American Society for Testing and Materials (ASTM) standard. To the authors’ knowledge, there is not a standard issued for these types of measurements. Additionally, filament diameter measurements were taken with both the integrated sensor of the 3Devo extruder and manually with a high-quality caliper.

### 2.6. Mechanical Characterization of the 3D-Printed Specimens

All the 3D-printed specimens manufactured were subjected to the same set of mechanical tests, following the corresponding international standards for testing. All tests (as mentioned five specimens for each case and each different type of test) were conducted at room temperature and humidity of about 55% (RH). The tests and the parameters with which they were performed are listed below:

1. Tensile test: Imada model MX2 (Northbrook, IL, USA), ASTM D638-02a elongation speed 10 mm/min, Type V (3.2 mm thickness).
2. Flexural test: Imada model MX2 (Northbrook, IL, USA), ASTM D790, elongation speed 10 mm/min, span 52 mm, three-point-bending.
3. Impact test: Terco MT 220 (Kungens Kurva, Sweden), ASTM D6110, release height 367 mm, Charpy Notched.
4. Microhardness measurements: Innova Test model 300 (Maastricht, The Netherlands), ASTM E384-17, Indentation 10 s, load 200 gF, Vickers microhardness test type

### 2.7. Morphological Characterization of the 3D-Printed Specimens with Scanning Electron Microscopy (SEM)

Scanning electron microscopy (SEM) (JEOL JSM 6362LV, Peabody, Massachusetts, Unites States, gold-sputtered specimens, 20 Kv) was utilized for the morphological characterization of the 3D-printed samples. Randomly selected tensile specimens, one from each different material prepared in the work, were examined for their morphological characteristics. Images were taken with different magnification levels from the side of the specimens to evaluate the layer fusion quality and determine possible defects in the 3DP structure. The fracture surface was also investigated, to reveal the fracture mechanism on the specimens. Additionally, energy dispersive x-ray analysis (EDX) was performed on non-sputtered samples, to verify the basic elements in each different nanomaterial.

### 2.8. Screening Test for the Antibacterial Performance of the Nanocomposites

The nanocomposites featuring the antibacterial additives investigated in the work (Cu and Cu_2_O) were anticipated to have biocidal performance. To verify such behavior from the prepared nanomaterials, a screening agar-well diffusion process [81] was conducted. Cylindrical specimens (12.5 mm in diameter, 4 mm in height) were 3D-printed with the corresponding nanomaterials in the work and they were assessed with this process against two bacteria, i.e., gram-negative Escherichia Coli (*E. coli*) and gram-positive Staphylococcus aureus (*S. aureus*). The bacteria were sourced from the local University Hospital’s microbiological laboratory following all the foreseen safety measures, and they were then identified by the local association of microbiologists before their use in the tests.

Specimens were placed in 85 mm in diameter Petri dishes (initially dried in an oven for 30 min, to remove humidity and numbered to distinguish the different nanomaterials tested) having each the appropriate for each bacterium growth agent (MC.2, C.010066 for *E. coli* and Chapman, C.010068 for *S. aureus*). A bacterium solution with natural serum was prepared for each bacterium and the solution was homogenized and spread in the growth agent of each Petri dish before the placement of the specimens. The bacteria concentration in the solution was adjusted using the McFarland 0.5 standard (the 0.5 McFarland turbidity standard is comparable to the density of a bacterial suspension with a 1.5 × 10^8^ CFU/mL) [82,83,84], which is based on the turbidity of the solution, and it is the most commonly used standard in clinical microbiology laboratory [84]. The exact concentration is not known, but it was the same for all specimens tested for both bacteria.

The process was optically examined, and a homogeneous spread was achieved. To ensure adequate bacteria populations in the solutions, the bacterium colonies were verified with an optical microscope before they were spread in the Petri dishes. Petri dishes with the specimens were subjected to 37 °C for 24 h in a laboratory oven. Afterward, the Petri dishes were optically examined to determine whether inhibition zones (IZs) were developed around the specimens. These IZs were optically measured, following the same process for each sample.

## 3. Results

### 3.1. Thermogravimetric Analysis (TGA) and Raman Spectra

**Figure 3:**
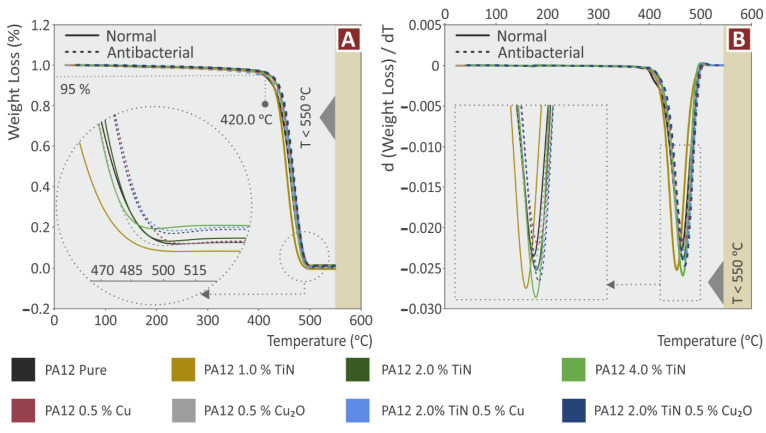
TGA curves: (**A**) weight (%) compared to temperature (°C); (**B**) rate of the mass degradation (dw/dT) compared to temperature (°C).

Figure 3 presents the weight loss and the weight loss rate compared to temperature curves for all the materials prepared and tested in the work. A rather similar response is observed in the graphs, indicating an insignificant repercussion in the thermal properties of the induced additives in the PA12 polymer (Figure 3A). A critical temperature for the materials is 420 °C, in which all the materials start to rapidly lose weight. This temperature verifies that the extrusion temperatures in the work do not cause any degradation in the materials. The remaining weight at the end of the measurement agrees with the filler loadings in all materials. Only the PA12/TiN 1.0 wt.% nanocomposite shows a slightly different response, with the degradation temperature shifted to lower temperatures. Still, differences are insignificant. The weight loss rate curves agree with the weight loss curves, with the materials showing the maximum weight loss rate at almost the same temperature and with similar weight loss values (Figure 3B). The PA12/TiN 4.0 wt.% nanocomposite showed the highest weight loss value, followed by the PA12/TiN 1.0 wt.%, which again exhibited the maximum weight loss rate at a slightly lower temperature. These results reveal the thermal properties of the nanocomposites prepared in the work and verify that the thermal stability of the PA12 polymer was maintained after the addition of the fillers, for all cases and fillers’ combinations studied.

**Figure 4:**
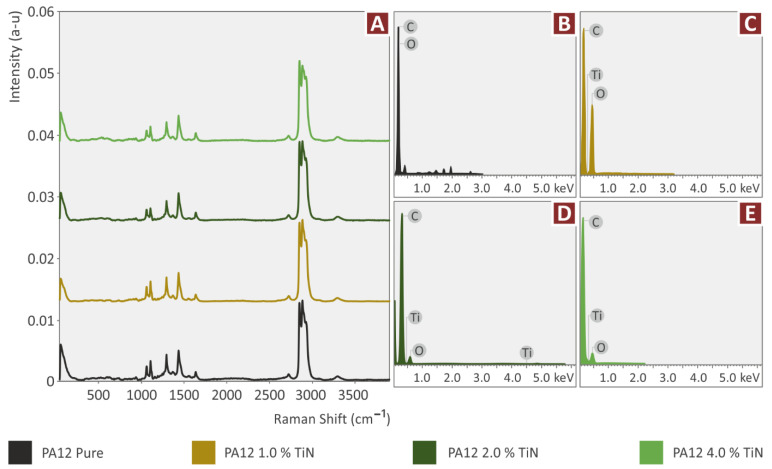
(**A**) Raman spectra, and EDS for (**B**) pure PA12, (**C**) PA12/TiN 1 wt.%, (**D**) PA12/TiN 2 wt.%, and (**E**) PA12/TiN 4 wt.%.

**Figure 5:**
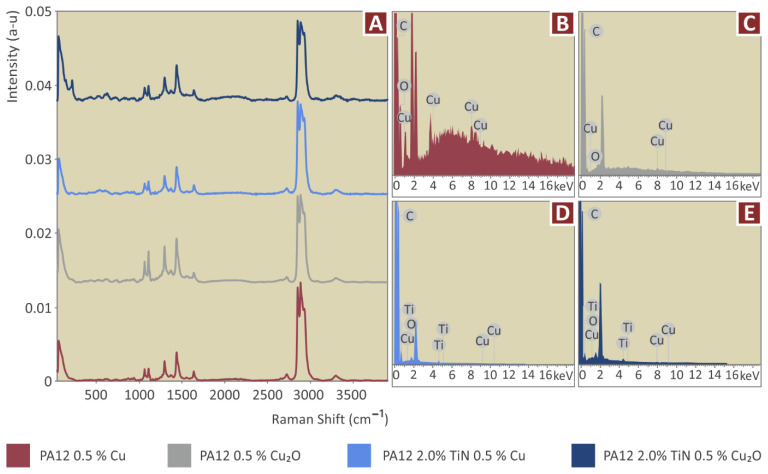
(**A**) Raman spectra curves, and EDS for (**B**) PA12/Cu 0.5 wt.%, (**C**) PA12/Cu_2_O 0.5 wt.%, (**D**) PA12/TiN 2.0 wt.%/Cu 0.5 wt.%, and (**E**) PA12/TiN 2.0 wt.%/Cu_2_O 0.5 wt.%.

In Figure 4 the major Raman peaks from PA12 Pure and PA12/TiN, at the various concentrations prepared in the work, are presented. Figure 5 presents the major Raman peaks for the nanocomposites prepared in the work containing the antibacterial additives (Cu and Cu_2_O). Clearly, C-O-C stretching was found at 1060, 1105, and 1293 cm^−1^. CH_2_ deformation and CH_2_ deformation were found at 1418 and 1441 cm^−1^ respectively. Lastly, CH_2_ symmetric stretching and deformation were identified at 1434 cm^−1^, 2850 cm^−1^, 2884 cm^−1^, and 2923 cm^−1^. Please see Table 1.

Adding TiN particles in PA12 presented a clear drop in the C-O-C bond stretching at 1060, 1105, and 1293 cm^−1^. Moreover, a drop in CH2 deformation was found at 1434 cm^−1^. Please see Table 2.

When Cu and Cu_2_O were added in PA12, there were no extra Raman lines identified. Interestingly, when Cu and Cu_2_O were added together with TiN in PA12, a small new Raman peak was presented at 1081 cm^−1^, referring to the C-N bond. Please see Table 3 and Figure 5.

### 3.2. Filament Quality Control

**Figure 6:**
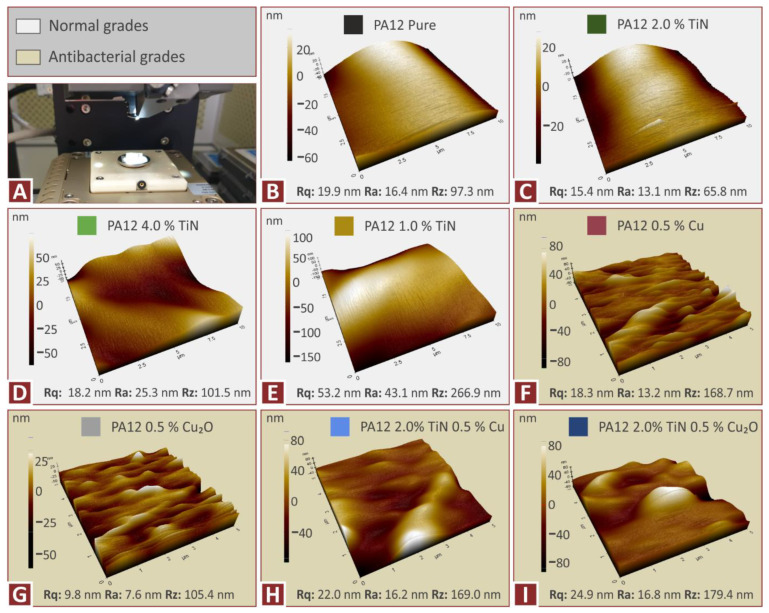
Filament quality control with AFM: (**A**) AFM setup, and PA12 (**B**) pure, (**C**) TiN 2 wt.%, (**D**) TiN 4 wt.%, (**E**) 1 wt.%, (**F**) Cu 0.5 wt.%, (**G**) Cu_2_O 0.5 wt.%, (**H**) TiN 2 wt.%, Cu 0.5 wt.%, (**I**) TiN 2 wt.%, Cu_2_O 0.5 wt.%.

Measurements taken on the produced filament for all the nanocomposites prepared in the work, i.e., with the integrated sensor of the 3devo extruder, which measures the diameter of the produced filament in real-time and with random manual measurements with a caliper, showed that the produced filament diameter was within acceptable limits, ensuring that they could be processed in the MEX 3D printer. The morphological investigation of the side surface of the filament with AFM (Figure 6) showed that the addition of the filler does not significantly affect the surface roughness and hence the quality of the surface, compared to pure PA12 polymer. Surface roughness measurement values do not significantly differ, as shown in the figure.

### 3.3. Mechanical Characterization Results from the 3D-Printed Specimens Testing

**Figure 7:**
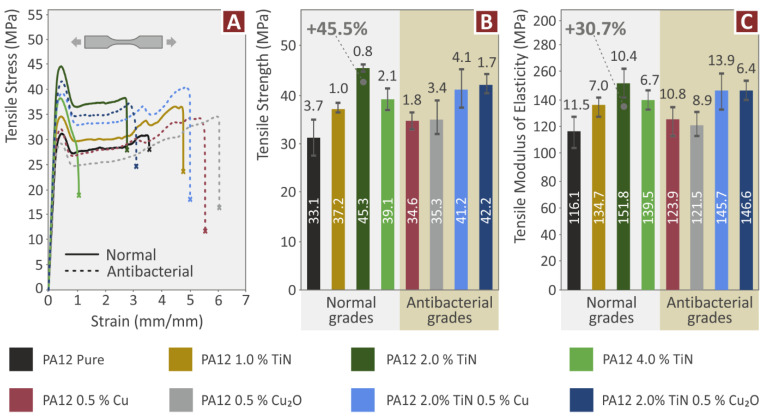
Tensile test results: (**A**) stress compared to strain curves, (**B**) average tensile strength and deviation (five samples), and (**C**) average tensile modulus of elasticity and deviation (five samples).

Specimens from all the nanocomposites prepared and pure PA12 were subjected to tensile testing, following the ASTM D638 standard. Figure 7A shows one randomly selected (from the five samples tested) stress compared to a strain graph from each different material. From the graphs, it is observed that nanocomposites containing only the TiN additive exhibit higher tensile strength and more brittle behavior, since they fail at lower strain values. Nanocomposites containing only the antibacterial additives (Cu and Cu_2_O) showed the more ductile behavior among the materials tested. Figure 7B depicts the tensile strength calculated for each material studied in the work. All the nanocomposites prepared in the work exhibit improved tensile strength compared to the pure PA12. The highest enhancement of 45.5% compared to the pure PA12 was achieved on the nanocomposite with 2 wt.% TiN loading, showing that the TiN additive can significantly enhance the tensile strength of the PA12. The nanocomposites containing only the antibacterial additives (Cu and Cu_2_O) also showed slightly improved mechanical performance in the tensile tests, showing that these additives not only induce antibacterial properties to the nanocomposites but, at the same time, they can also slightly enhance the tensile properties of the materials. The nanomaterials with binary inclusions exhibited significantly enhanced tensile strength compared to the pure PA12. Tensile strength values were close but slightly lower than the corresponding nanocomposite containing only the TiN additive at 2 wt.% loading, which exhibited the highest tensile strength in the tests. The nanocomposites with binary inclusions had the same TiN loading (2 wt.%) and showed a similar, but slightly reduced mechanical response. So, the addition of the second filler influenced the tensile response of the nanocomposites. This is probably because the second filler affected the development of the same nanoparticles network, being developed in the nanocomposite with only the TiN additive. Still, both the nanocomposites with binary inclusions developed about 7–9% lower tensile strength that the nanocomposite with the highest tensile strength in the work. This tensile strength is still about 40% higher than pure PA12 polymer. The tensile modulus of elasticity results presented in Figure 7C shows the exact same pattern as the tensile strength results. The highest tensile modulus of elasticity is reported again in the nanocomposite with 2 wt.% TiN loading, with a nanocomposite being 30.7% stiffer than the pure PA12 material. All the materials tested again had increased tensile modulus of elasticity values, compared to the pure PA12 polymer, showing that the addition of these specific fillers with the methodology followed in the work, not only increases the tensile strength of the materials, but also it makes them stiffer. As mentioned above, the nanocomposites containing only the TiN filler showed ductile behavior, while all the other nanocomposites of the work, had increased tensile performance, without compromising the ductile behavior of the PA12 polymer.

**Figure 8:**
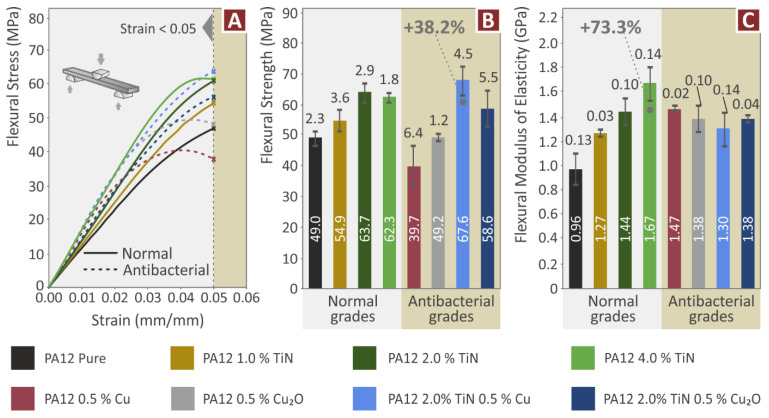
Flexural tests (experiments were stopped at strain 5%, following the ASTM D790 standard): (**A**) stress compared to strain, (**B**) average flexural strength and deviation (five samples), and (**C**) average flexural modulus of elasticity and deviation (five samples).

The flexural test results (Figure 8) follow the same pattern as the tensile test results in the nanocomposites containing only TiN as filler. The flexural strength is improved compared to the pure PA12 polymer in all the nanocomposites containing only the TiN additive (Figure 8B). In the nanocomposites with the antibacterial agents, the response of the nanocomposites differs. The nanocomposite containing only the Cu additive has an inferior response in the flexural test to the pure PA12 polymer. Since its flexural strength is adequate for use in applications, its tensile strength is enhanced compared to the pure PA12 polymer and it is expected to have antibacterial performance, it has enough merit as a nanocomposite itself. The nanocomposite containing only the Cu_2_O additive has similar flexural strength to the pure PA12 polymer, with the addition of the filler not affecting the flexural strength. Its merit is the same as the nanocomposite containing only the Cu additive. Such differences in the response of the materials in the tensile and the flexural tests are expected, attributed to the different loading and the stresses (tensile vs. tensile and compressive) developed in the parts because of the difference in the loads. This combined with the anisotropy of the 3D parts also contributes to the different responses of the samples in the different mechanical tests.

The nanocomposites with binary inclusions showed enhanced performance in the flexural tests, with the nanocomposite containing 2 wt.% TiN and 0.5 wt.% Cu exhibited the highest flexural strength among the materials tested, which was a 38.2% increase when compared to the pure PA12 polymer. The nanocomposite with 2 wt.% TiN and 0.5 wt.% Cu_2_O exhibited about 20% improved flexural strength compared to the pure PA12 polymer. Still, it had about 8% lower flexural strength than the nanocomposite containing only the TiN additive at the same loading. The addition of the fillers has a positive effect on the flexural modulus of elasticity (Figure 8C), making the nanocomposites stiffer in the flexural tests for all cases studied. The highest improvement in the flexural modulus of elasticity, of 73.3% compared to the pure PA12 polymer, is reported in the nanocomposite with the TiN filler at 4 wt.% concentration. The nanocomposites containing only the antibacterial additives (Cu and Cu_2_O) showed stiffness in the flexural tests similar to that of the nanocomposites containing only the TiN filler. The nanocomposites with binary inclusions had a slightly inferior performance regarding their stiffness in the flexural tests, still, their response was 35–40% higher than the pure PA12 polymer.

**Figure 9:**
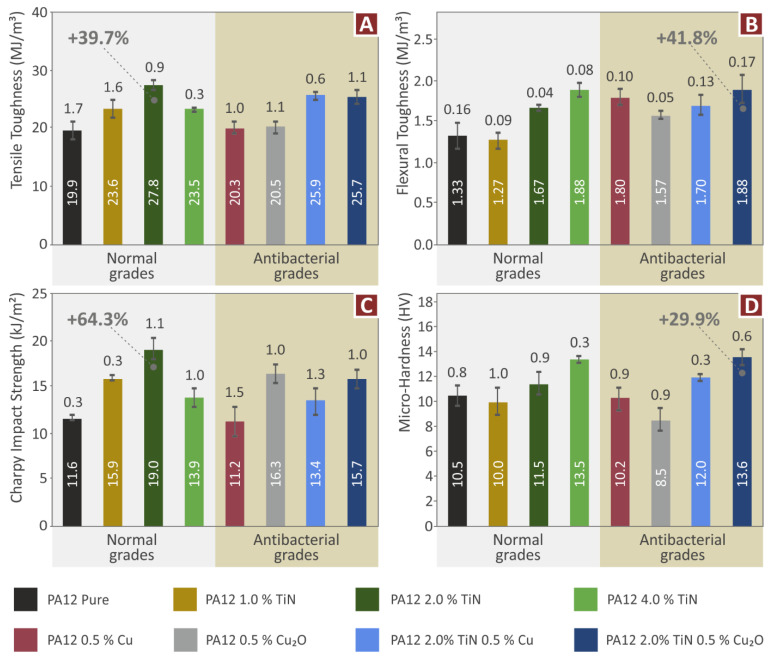
(**A**) average tensile toughness (MJ/m^3^) and deviation (five samples), (**B**) average flexural toughness (MJ/m^3^), and deviation (five samples), (**C**) impact strength (kJ/m^2^) and deviation (five samples), (**D**) Vickers microhardness and deviation (five samples).

The tensile and flexural toughness values are presented in Figure 9A,B respectively. These values show the absorbed energy of the materials during the corresponding tests and are calculated as the integral of the respective stress compared to stain graphs. Therefore, these values do not necessarily follow the trend of the corresponding strength values, as they are affected by the ductileness of the material. In both tests (tensile and flexural) all the nanocomposites had increased toughness compared to the pure PA12 polymer. In the tensile test, the highest tensile toughness is reported for the nanocomposite containing only the TiN additive at a concentration of 2 wt.% (39.7% higher than the pure PA12 polymer). In the flexural test, the highest flexural toughness is reported for the nanocomposite containing binary inclusions, i.e., TiN 2 wt.% and Cu_2_O 0.5 wt.% (41.8% higher than the pure PA12 polymer). Overall, the nanocomposites with binary inclusions follow the trend of the corresponding test results.

The Charpy’s impact test results (Figure 9C) show that all the materials tested had improved impact strength compared to the pure PA12 polymer, verifying the enhancement effect of the additives studied. The only exception in this test was the nanocomposite containing only the Cu additive at 0.5 wt.% which did not show improvement compared to the pure PA12 polymer, with the impact strength values being similar to the pure polymer. Again, the differences in the materials’ responses compared to the other mechanical tests, can be attributed to the nature of the impact test and the differences in the loading and the developed stresses in the samples, in combination with the anisotropic nature of the MEX 3D-printed structures. The highest impact strength is reported for the nanocomposites containing the TiN additive at 2 wt.% concentration (64.3% higher impact strength compared to the pure PA12 polymer). The second highest impact strength is reported for the nanocomposites containing the Cu_2_O additive at 0.5 wt.% (about 40% higher impact strength compared to the pure PA12 polymer). The corresponding nanocomposite with binary inclusions, containing TiN 2.0 wt.% and Cu_2_O 0.5 wt.% had a rather similar performance in the impact test (about 36% higher impact strength compared to the pure PA12 polymer and about 20% lower impact strength than the highest reported in the work). The nanocomposite with binary inclusions, containing TiN 2.0 wt.% and Cu 0.5 wt.% had decreased impact strength compared to the nanocomposite containing only the TiN additive at the same concentration of 2 wt., but significantly higher impact strength (about 20%) than the nanocomposite containing only the Cu additive at 0.5 wt.% concentration.

The Vickers microhardness results (Figure 9D) show that the addition of TiN increases the Vickers microhardness of the nanocomposites compared to the pure PA12 polymer, at the highest concentrations studied. At low concentrations, it does not affect the Vickers microhardness values. Nanocomposites containing only the antibacterial additives (Cu and Cu_2_O) had decreased response in the Vickers microhardness measurements. Nanocomposites with binary inclusions reported improved Vickers microhardness measurements. The nanocomposite containing TiN 2.0 wt.% and Cu_2_O 0.5 wt.% had the highest Vickers microhardness among the materials tested, 29.9% higher than the pure PA12 polymer. The nanocomposite containing TiN 2.0 wt.% and Cu 0.5 wt.% had increased Vickers microhardness compared to the corresponding nanocomposite containing only the TiN additive, with the same concentration (2 wt.%). These results show a positive effect of the binary inclusions in the Vickers microhardness of the materials tested herein.

### 3.4. Morphological Characterization of the 3D-printed Specimens with Scanning Electron Microscopy (SEM)

**Figure 10:**
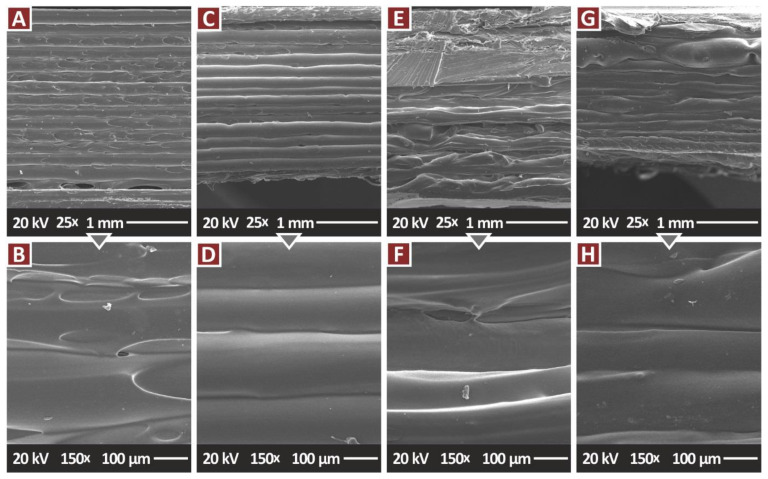
SEM images, tensile specimens side surface: (**A**) PA12 Pure 25×, (**B**) PA12 Pure 150×, (**C**) PA12/TiN 1.0 wt.% 25×, (**D**) PA12/TiN 1.0 wt.% 150×, (**E**) PA12/TiN 2.0 wt.% 25×, (**F**) PA12/TiN 2.0 wt.% 150×, (**G**) PA12/TiN 4.0 wt.% 25×, (**H**) PA12/TiN 4.0 wt.% 150×.

Figure 10 presents side surface images at two magnifications of the pure PA12 and the nanocomposites containing only the TiN additive, in all the filler concentrations prepared in the work. These images were taken on tensile samples, as mentioned. In the pure PA12 (Figure 10A,B) the layer fusion is excellent, and the layers are formed as expected for a MEX 3D printing structure. Few voids are visible in one specific layer. This is probably a random phenomenon in this specific sample. In the nanocomposite with TiN 1 wt.% (Figure 10C,D) a similar well-formed 3D-printed is depicted, with no visible voids or defects. As the TiN concentration in the nanocomposite increases, the fusion between the layers is maintained at a high level, but the layers are not that well-formed, as presented for the nanocomposite with 2 wt.% loading. Still, no voids are visible. The images from the nanocomposites with 4 wt.% loading have a similar appearance to the 2 wt.% loading nanocomposites, indicating that the built structure was not affected by the TiN loading increase in the nanocomposite.

**Figure 11:**
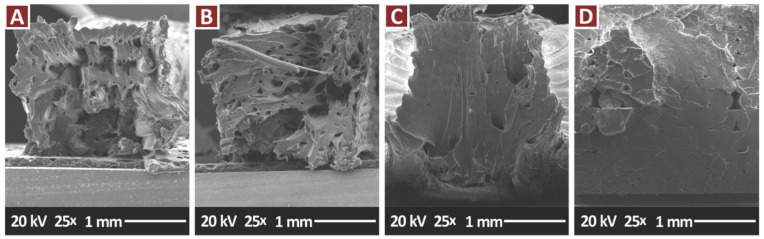
SEM images, tensile specimens fracture surface at 25×: (**A**) PA12 Pure, (**B**) PA12/TiN 1.0 wt.%, (**C**) PA12/TiN 2.0 wt.%, (**D**) PA12/TiN 4.0 wt.%.

Figure 11 presents the fracture areas of the pure PA12 and the nanocomposites containing only the TiN additive, in all the filler concentrations prepared in the work. These images agree with the tensile test results, in which the increase of the TiN concentration in the nanocomposites increases the brittleness of the samples. Pure PA12 (Figure 11A) and PA12/TiN with 1 wt.% (Figure 11B) fracture areas show deformation and a collapse of the sample during its failure and cavities and voids in the fracture surface were created. The corresponding images from the nanocomposites with 2 wt.% (Figure 11C) and 4 wt.% (Figure 11D) TiN concentration present a brittle fracture surface, with minimum deformation, with the phenomenon being intense at the highest filler loading.

**Figure 12:**
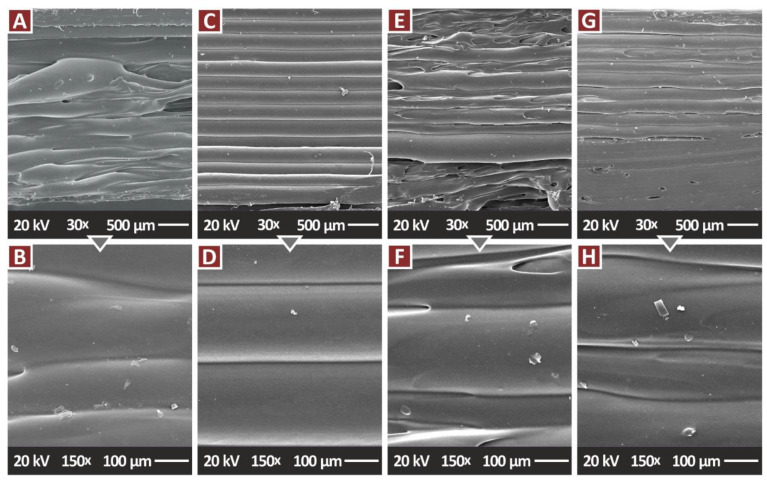
SEM images, tensile specimens with antibacterial properties, side surface: (**A**) PA12/Cu 0.5 wt.% 30×, (**B**) PA12/Cu 0.5 wt.% 150×, (**C**) PA12/Cu_2_O 0.5 wt.% 30×, (**D**) PA12/Cu_2_O 0.5 wt.% 150×, (**E**) PA12/Cu 0.5 wt.%/TiN 2.0 wt.% 30×, (**F**) PA12/Cu 0.5 wt.%/TiN 2.0 wt.% 150×, (**G**) PA12/Cu_2_O 0.5 wt.%/TiN 2.0 wt.% 30×, (**H**) PA12/Cu_2_O 0.5 wt.%/TiN 2.0 wt.% 150×.

**Figure 13:**
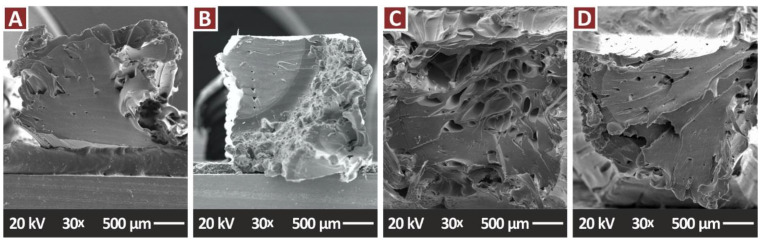
SEM images, tensile specimens with antibacterial properties, fracture surface at 30×: (**A**) PA12/Cu 0.5 wt.%, (**B**) PA12/Cu_2_O 0.5 wt.%, (**C**) PA12/Cu 0.5 wt.%/TiN 2.0 wt.%, (**D**) PA12/Cu_2_O 0.5 wt.%/TiN 2.0 wt.%.

Figure 12 presents side surface images at two magnifications of the nanocomposites containing the antibacterial additives. In the case of PA12/Cu_2_O 0.5 wt.% (Figure 12C,D) layer fusion is excellent, and the layers are formed as expected for a MEX 3D printing structure. In all the other test samples, the fusion between the layers is maintained at a high level, but the layers are not that well-formed, and a small number of defects can be observed in the images. This is more intense in the nanocomposites with binary inclusions, with the PA12/TiN/Cu_2_O sample (Figure 12G,H) having a better-formed layer structure and less visible voids than the PA12/TiN/Cu nanocomposite (Figure 12E,F). The fracture surface magnifications of the nanocomposites containing the antibacterial additives are presented in Figure 13. Again, the fracture surfaces agree with the tensile test results, with the single additive nanocomposites (PA12/Cu—Figure 13A, PA12/Cu_2_O Figure 13B) showing a more brittle behavior with minimum deformation visible in the images. The binary inclusions nanocomposites (PA12/TiN/Cu—Figure 13C, PA12/TiN/Cu_2_O Figure 13D) show a more ductile behavior, with deformation visible in the fracture areas, especially in the PA12/TiN/Cu nanocomposite.

**Figure 14:**
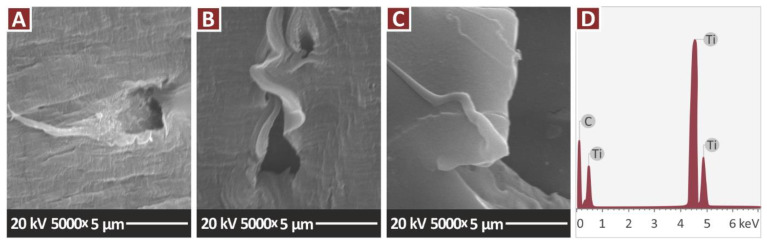
SEM images, tensile specimens, fracture surface at 5000×: (**A**) PA12/TiN 1.0 wt.%, (**B**) PA12/TiN 2.0 wt.%, (**C**) PA12/TiN 4.0 wt.%, (**D**) EDS on the area captured on the PA12/TiN 4.0 wt.%.

**Figure 15:**
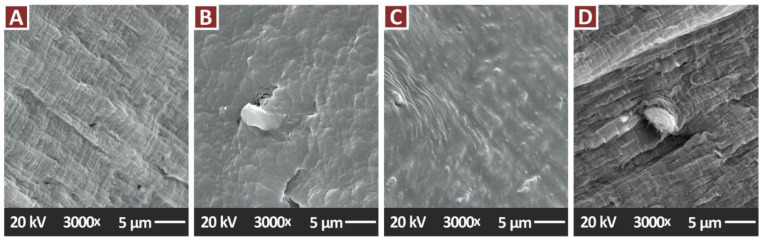
SEM images, tensile specimens with antibacterial properties, fracture surface at 3000×: (**A**) PA12/Cu 0.5 wt.%, (**B**) PA12/Cu_2_O 0.5 wt.%, (**C**) PA12/Cu 0.5 wt.%/TiN 2.0 wt.%, (**D**) PA12/Cu_2_O 0.5 wt.%/TiN 2.0 wt.%.

Figure 14A–C present higher magnification images of 5000× of the nanocomposites containing only the TiN additive, in all the filler concentrations prepared in the work. These images were taken to examine the nanocomposites for possible agglomerations of the filler in the matrix. No agglomerations were found when examining the fracture area of the samples at this magnification level. At this magnification level EDS was performed (Figure 14D) and the elements of the nanocomposites were verified, while no additional elements were identified with this process. Figure 15 presents higher magnification images of 3000× of the nanocomposites containing the antibacterial additives. Again, these images were taken to examine the nanocomposites for possible agglomerations of the filler in the matrix. Minimum to no agglomerations were located in the samples, indicating that a good dispersion of the additives in the matrix material was achieved with the method followed in the work for their preparation.

### 3.5. Screening Test for the Antibacterial Performance of the Nanocomposites

**Figure 16:**
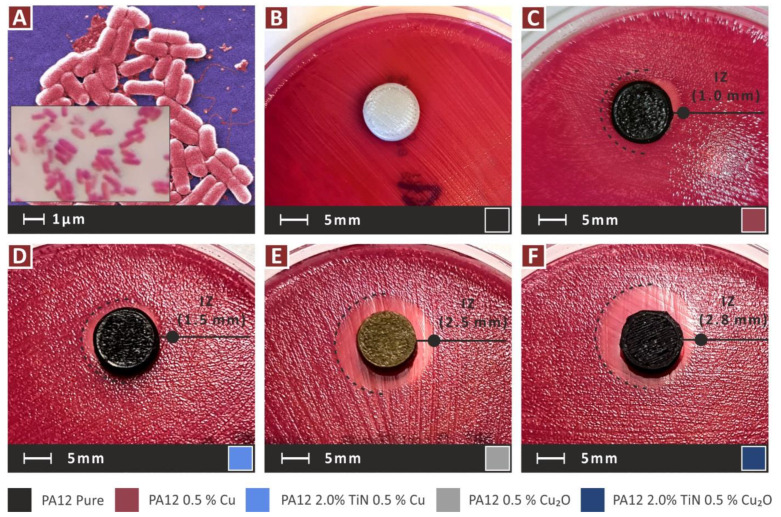
*E. coli* bacterium biocidal performance of the nanocomposites: (**A**) *E. coli*, and PA12 (**B**) pure, (**C**) 0.5 wt.% Cu, (**D**) 0.5 wt.% Cu, 2.0 wt.% TiN, (**E**) 0.5 wt.% Cu_2_O, and (**F**) 0.5 wt.% Cu_2_O, 2.0 wt.% TiN.

**Figure 17:**
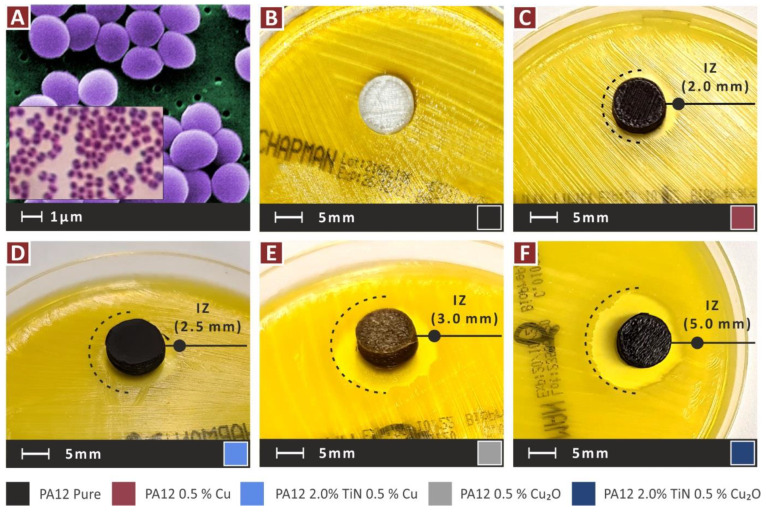
*S. aureus* bacterium biocidal performance of the nanocomposites: (**A**) *S. aureus*, and PA12 (**B**) pure, (**C**) 0.5 wt.% Cu, (**D**) 0.5 wt.% Cu, 2.0 wt.% TiN, (**E**) 0.5 wt.% Cu_2_O, and (**F**) 0.5 wt.% Cu_2_O, 2.0 wt.% TiN.

Figure 16 presents the inhibition zones (IZs) developed during the screening agar-well diffusion tests, against gram-negative *E. coli*. Figure 16B presents a pure PA12 sample, in which, as expected, no IZ has been developed. Apart from the pure PA12, only the nanocomposites with the antibacterial additives were tested (Cu and Cu_2_O). As is shown, all the nanocomposites exhibited antibacterial properties. The PA12/Cu_2_O nanocomposite developed the wider IZ among the nanomaterials tested, with the PA12/TiN/Cu_2_O nanocomposite being the second best in the test. This indicates that Cu_2_O showed better antibacterial performance against the gram-negative *E. coli* than the Cu nanocomposite. Figure 17 shows the corresponding results against gram-positive *S. aureus*. A similar response is observed, with the pure PA12, as expected, not developing IZ, and the nanocomposites with the Cu additive developing narrower IZs than the nanocomposites with the Cu_2_O additive. So, Cu_2_O showed better antibacterial performance against the gram-positive *S. aureus*, too, and overall, a better antibacterial performance than Cu for the two bacteria tested. In the case of gram-positive *S. aureus*, the PA12/TiN/Cu_2_O nanocomposite developed wider IZ than the PA12/Cu_2_O nanocomposite, showing that the addition of the TiN filler not only did not compromise the antibacterial properties of the nanocomposite but, on the contrary, it enhanced, in this case, the antibacterial performance of the nanocomposite.

## 4. Discussion

**Figure 18:**
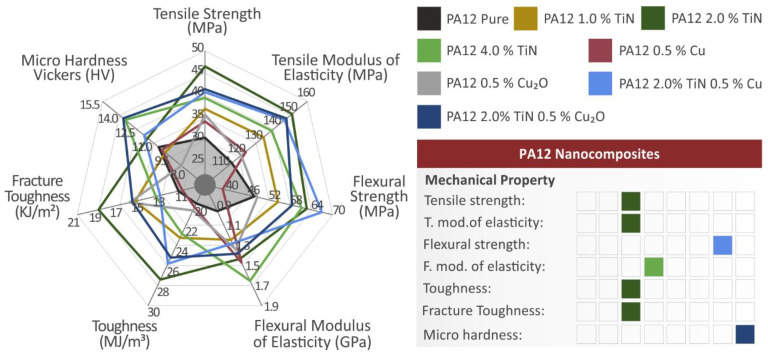
A spider graph summarizing the mechanical characterization process results. The shaded area depicts the performance of the pure PA12 polymer. The material exhibiting the highest results in each mechanical test is depicted on the right side of the figure.

In this work, the aim of developing multifunctional nanocomposites with enhanced mechanical properties and antibacterial properties for MEX 3DP was achieved. TiN aimed to enhance the mechanical properties of the nanocomposites, while two different additives, i.e., Cu and Cu_2_O, were investigated for inducing antibacterial properties to the nanocomposites. All additives were tested individually for their effect on the PA12 matrix material, with the development of single inclusion nanocomposites, in each case. All had a positive effect on the mechanical properties of the PA12 polymer. Then nanocomposites with binary inclusions, combing TiN and one of the two antibacterial agents (Cu and Cu_2_O) were developed. Nanomaterials with binary inclusions showed improved mechanical response compared to the pure PA12 polymer. In the tensile tests, the nanomaterials with binary inclusions showed lower tensile strength than the corresponding nanocomposite featuring only the TiN additive with the same loading, as mentioned, and analyzed in the results section. In the flexural tests, the PC/TiN/Cu nanocomposite had the highest flexural strength among the materials tested. Overall, the enhancement with the addition of these additives in the PA12 polymer was achieved. The mechanical test results are summarized in Figure 18.

No similar nanocomposites are available in the literature for AM or in bulk form with which to compare and evaluate the results of the study. Nanocomposites using polycarbonate (PC) as a matrix material and TiN in MEX 3DP show a similar enhancement effect, by the addition of the TiN filler in the PC matrix material [56]. Additionally, nanomaterials with binary inclusions for MEX 3DP, using Polylactic Acid (PLA) as the matrix material and Cu_2_O as one of the two fillers, also had a similar trend to the results of this study [35]. Regarding the antibacterial performance of the developed nanocomposites featuring the antibacterial additives (Cu and Cu_2_O), all the nanocomposites developed had biocidal properties at least for the two bacteria tested, i.e., gram-negative *E. coli* and gram-positive *S. aureus*. Still, the nanocomposites with the Cu_2_O additive showed superior biocidal performance compared to the nanocomposites with the Cu additive.

In this work, the possibility of developing nanocomposites with multi-functional behavior, with a rigorous and effective process, which can have industrial merit and can be easily adapted in such environments is investigated. In the medical field, medical-grade 3D-printed parts can be employed in several types of applications. The most common application of such materials is in surgical guides, while they have also been used in orthopedics and surgical planning [88]. The antibacterial properties of the nanocomposites developed herein are an additional asset, highly sought after in the medical field [89].

The preparation of the nanocomposites consisted of steps aiming to achieve good dispersion of the filler in the matrix material, with simple processes and equipment. First, the raw materials were vigorously mixed in a high-power blender for sufficient time. Then, a first extrusion process aimed to provide an initial distribution of the fillers in the matrix material. Afterward, the produced nanocomposites’ filament was shredded to pellets and underwent a second extruding process, in a special MEX 3D printing extruder, with a capability for materials and additives mixing, due to the geometry of its screw, according to its manufacturer. With this process, no agglomerations were located in the examination of the 3D-printed specimen surfaces with SEM and EDS, in which higher magnifications were employed in the SEM, for the determination of the nanocomposites’ elements. Higher peaks for the additive elements would indicate a high concentration of the specific element in the EDS observation region. Additionally, the deviations in the mechanical tests are within acceptable limits, indicating a similar material composition in all cases tested. These data indicate a good dispersion of the additives in the matrix material with the process followed in this work.

Figure 11 and Figure 13 present SEM images from the fracture surfaces of tensile test samples. As expected, fracture surfaces are highly deformed, especially considering that the PA12 polymer has that type of behavior. Filament strands in the 3D printing structure are highly deformed before the failure of the part, creating internal cavities in the specimens in the fracture area.

From the TGA tests conducted in all the materials prepared in the work, it was found that the thermal stability of the matrix material was maintained for all the additives and additives combinations tested in the work. Also, the temperatures set in the extrusion processes do not cause any degradation in the materials. The EDS analysis verified the elements in the nanocomposites, while no additional unexpected elements were identified in the process (Figure 4B–E and Figure 5B–E). It should be noted that EDS graphs and peaks are dependent on the observation region of the samples and may differ from one region to another, especially considering that EDS was performed in a high magnification SEM image. EDS observations were acquired from randomly selected regions on the samples.

The morphological characteristics of the 3D-printed samples prepared with the nanocomposites of this work were investigated with SEM. The 3D printing quality was evaluated, and the fracture mechanism was revealed for the tensile test samples. Higher magnification images were also taken in an attempt to locate possible agglomerations in the nanocomposites. Minimum to no agglomerations was located. This combined with the mechanical test results, in which in all tests the calculated deviation was within acceptable limits, indicates that NPs formed a well-distributed network, with good dispersion, with the process followed in the work for their preparation.

The development of such composites with multifunctional performance is also a cost-effective process since the only additional cost in the process is the cost of the additives used. Considering that the commercial filament costs about ten times the cost of the raw materials in MEX 3D printing for most of the polymers, it can be extracted that the main cost in the MEX 3DP polymers is the preparation process cost and not the cost of the raw material itself. The PA12 polymer in the form it was sourced in the current work costs about 0.027 EUR/gr. TiN additive costs about 0.9 EUR/gr, Cu nanopowder about 2.4 €/gr, and Cu_2_O about 2.6 €/gr (for a 100 gr package). Considering these prices, the cost per gram is increased from 0.027 EUR/gr for the pure PA12 polymer to 0.057 EUR/gr for the nanocomposite with 2 wt.% TiN and 0.5 wt.% Cu. Considering the 1/10 ratio for the raw materials in the total cost, this is not a significant increase in the total cost. Additionally, these costs can be significantly reduced for industrial-scale material quantities.

## 5. Conclusions

In this work, for the first-time multifunctional nanocomposites for MEX 3DP were prepared, featuring enhanced mechanical performance and antibacterial properties. To achieve that, nanocomposites with binary inclusions were produced with a thermomechanical process, using PA12 as the matrix material. One additive (TiN) was used for the mechanical enhancement and a second one (Cu or Cu_2_O) with known biocidal performance, induced the antibacterial properties in the nanocomposites. The produced nanocomposites were thoroughly investigated for their mechanical, thermal, spectroscopic, antibacterial, and morphological characteristics. It was found that they exhibited improved mechanical response compared to PA12 polymers, their thermal stability of the matrix material was not affected by the introduction of the fillers, and all the nanocomposites with antibacterial additives had biocidal properties in the screening tests conducted in the work. It was verified that TiN can enhance the mechanical properties of the PA12 polymer. Although both antibacterial additives (Cu or Cu_2_O) induced such properties in the nanocomposites, the performance of Cu_2_O in these tests was higher than that of Cu. No significant processability issues were faced during the preparation of the nanocomposites and the 3D-printed samples, indicating that the process can be easily adapted in larger-scale industrial environments. The nanocomposites developed herein are cost-effective, as analyzed above. Overall, among the nanocomposites produced with antibacterial properties, the nanocomposite with 2 wt.% TiN and 0.5 wt.% Cu_2_O showed the most enhanced response in both the mechanical and the antibacterial tests. Future work, additional and more advanced antibacterial tests can be conducted, while the process can be further optimized for industrial-scale applications.

## Figures and Tables

**Table 1:** Major Raman peaks identified and their related assignments.

Wavenumber (cm^−1^)	Raman Peak Assignment
1060	C-O-C stretching [85]
1105	C-O-C stretching [85]
1293	C-O-C stretching [85]
1434	CH_2_ deformation [85,86]
2850	CH_2_ symmetric stretching [87]
2884	CH_2_ symmetric stretching [87]
2923	CH_2_ asymmetric stretching [87]

**Table 2:** Raman peak differences, as they were identified.

Wavenumber (cm^−1^)	Raman Peak Assignment	Change
1060	C-O-C stretching [85]	Drop
1105	C-O-C stretching [85]	Drop
1293	C-O-C stretching [85]	Drop
1434	CH_2_ deformation [85,86]	Drop

**Table 3:** Raman peak differences, identified when Cu and/or Cu_2_O particles are added to PA12.

Wavenumber (cm^−1^)	Raman Peak Assignment	Change
1081	C-N bond	Small peak presented in Cu/TiN and Cu_2_O/TiN mixtures
1105	C-O-C stretching [85]	Increase in the PA12 with Cu_2_O sample

## Data Availability

The data presented in this study are available upon request from the corresponding author.
